# Red Cell Distribution Width (RDW), Platelets and Platelet Index MPV/PLT Ratio as Specific Time Point Predictive Variables of Survival Outcomes in COVID-19 Hospitalized Patients

**DOI:** 10.3390/jcm14155381

**Published:** 2025-07-30

**Authors:** Despoina Georgiadou, Theodoros Xanthos, Veroniki Komninaka, Rea Xatzikiriakou, Stavroula Baka, Abraham Pouliakis, Aikaterini Spyridaki, Dimitrios Theodoridis, Angeliki Papapanagiotou, Afroditi Karida, Styliani Paliatsiou, Paraskevi Volaki, Despoina Barmparousi, Aikaterini Sakagianni, Nikolaos J. Tsagarakis, Maria Alexandridou, Eleftheria Palla, Christos Kanakaris, Nicoletta M. Iacovidou

**Affiliations:** 1 Hematology Department, Sismanoglio-A. Flemig, General Hospital of Attica, 15127 Athens, Greece; 2 School of Health Sciences, University of West Attica, 12243 Athens, Greece; 3 Hematology Laboratory, G. Gennimatas, General Hospital of Attica, 11527 Athens, Greece; v.komninaka@gna-gennimatas.gr (V.K.); nikolaostsagarakis@gmail.com (N.J.T.); 4 Hematology Laboratory, Sismanoglio, General Hospital of Attica, 15126 Athens, Greece; aimatologiko@sismanoglio.gr; 5 Medical School, National Kapodistrian University of Athens, 11528 Athens, Greece; sbaka@med.uoa.gr; 6 2nd Department of Pathology, “Attikon” Hospital, National and Kapodistrian University of Athens, 12462 Athens, Greece; apouliak@med.uoa.gr; 7 Internal Medicine Department, Sismanoglio-A. Flemig, General Hospital of Attica, 15127 Athens, Greece; kspyridaki@yahoo.gr; 8 Hematology and Biochemistry Department, Konstantopouleio-Patision Hospital, 14233 Athens, Greece; dimdrteo@gmail.com (D.T.); elefthepal@gmail.com (E.P.); xristosxrisoba@hotmail.com (C.K.); 9 Hematological & Biochemical Department, Laiko Hospital, 11527 Athens, Greece; agpana@med.uoa.gr; 10 Hematology Laboratory, Evaggelismos Hospital, 10676 Athens, Greece; a.karidaevag@yahoo.com (A.K.); maria.1485@hotmail.com (M.A.); 11 2nd Department of Obstetrics & Gynecology, Aretaieio Hospital, National and Kapodistrian University of Athens, 11528 Athens, Greece; stpaliatsiou@yahoo.gr; 12 Department of Neonatology, Aretaieio Hospital, National and Kapodistrian University of Athens, 11528 Athens, Greece; v.volaki@hotmail.com (P.V.); niakobid@med.uoa.gr (N.M.I.); 13 Haematology Laboratory, Alexandra Hospital, 11528 Athens, Greece; aimatologiko@hosp-alexandra.gr; 14 Intensive Care Unit, Sismanoglio General Hospital of Attica, 15126 Athens, Greece; sakagianni@sismanoglio.gr

**Keywords:** COVID-19 disease mortality, red cell distribution width, RDW, platelets, PLT, platelet index MPV/PLT ratio, thrombogenicity index MPV/PLT ratio

## Abstract

**Background**: COVID-19-associated coagulopathy (CAC) is a complex condition, with high rates of thrombosis, high levels of inflammation markers and hypercoagulation (increased levels of fibrinogen and D-Dimer), as well as extensive microthrombosis in the lungs and other organs of the deceased. It resembles, without being identical, other coagulation disorders such as sepsis-DIC (SIC/DIC), hemophagocyte syndrome (HPS) and thrombotic microangiopathy (TMA). Platelets (PLTs), key regulators of thrombosis, inflammation and immunity, are considered an important risk mediator in COVID-19 pathogenesis. Platelet index MPV/PLT ratio is reported in the literature as more specific in the prognosis of platelet-related systemic thrombogenicity. Studies of MPV/PLT ratio with regards to the severity of COVID-19 disease are limited, and there are no references regarding this ratio to the outcome of COVID-19 disease at specific time points of hospitalization. The aim of this study is to evaluate the relationship of COVID-19 mortality with the red cell distribution width–coefficient of variation (RDW-CV), platelets and MPV/PLT ratio parameters. **Methods**: Values of these parameters in 511 COVID-19 hospitalized patients were recorded (a) on admission, (b) as mean values of the 1st and 2nd week of hospitalization, (c) over the total duration of hospitalization, (d) as nadir and zenith values, and (e) at discharge. **Results**: As for mortality (survivors vs. deceased), statistical analysis with ROC curves showed that regarding the values of the parameters on admission, only the RDW-CV baseline was of prognostic value. Platelet parameters, absolute number and MPV/PLT ratio had predictive potential for the disease outcome only as 2nd week values. On the contrary, with regards to disease severity (mild/moderate versus severe/critical), only the MPV/PLT ratio on admission can be used for prognosis, and to a moderate degree. On multivariable logistic regression analysis, only the RDW-CV mean hospitalization value (RDW-CV mean) was an independent and prognostic variable for mortality. Regarding disease severity, the MPV/PLT ratio on admission and RDW-CV mean were independent and prognostic variables. **Conclusions**: RDW-CV, platelets and MPV/PLT ratio hematological parameters could be of predictive value for mortality and severity in COVID-19 disease, depending on the hospitalization timeline.

## 1. Introduction

By the end of 2019, the first reports of an unknown respiratory infection, in several cases with a fatal outcome, emerged in the city of Wuhan, China. The cause was very quickly identified as the novel coronavirus, SARS-CoV-2, and the World Health Organization (WHO) declared the infection to be COVID-19, a Public Health Emergency of International Concern. By the beginning of March 2020, more than 90,000 people had been infected and at least 3100 deaths had been recorded [1].

As information kept occurring concerning the virus, COVID-19 infection and therapeutic management, research on clinical and laboratory markers related to the prognosis of severity and outcome of the disease was added.

Even prior to the COVID-19 era, in Perlstein’s large-scale prospective community cohort study (NHANES III), the red cell index RDW, that accounts for erythrocyte anisocytosis, was reported as a predictor of all-cause mortality, independent of CRP, and not substantially affected by anemia or lack of hematinics [2]. In addition, it had prognostic importance in cardiovascular disease (CAD) [3,4,5,6,7,8,9,10,11,12,13,14,15,16,17,18,19,20], thromboembolism [3,21,22,23], chronic obstructive pulmonary disease (COPD) [24], cancer [2,25,26,27,28], kidney disease [29], liver disease [30,31,32], diabetes mellitus [33,34,35] and sepsis [36,37,38]. In the explosion of publications for SARS-CoV-2, there has been extensive reference to the relationship of RDW with increased COVID-19 mortality to a statistically significant degree [39,40,41,42,43,44].

Moreover, since platelets are key regulators of thrombosis, inflammation and immunity, they become important mediators of COVID-19 pathogenesis [45,46]. Regarding the platelets in the meta-analysis by Jiang et al., in 25 studies despite high heterogeneity (j2 88.9–91.8%), in a total of 7613 COVID-19 patients up to April 2020, it appears that patients with severe disease had lower platelet values. In addition, non-survivors had much lower platelets than survivors, rendering thrombocytopenia as a possible risk factor for progression to a more severe disease [47].

With regards to platelet indices related to platelet size, it is known that increased mean platelet volume (MPV) is associated with various prothrombotic diseases, as large platelets represent new and just released ones, rich in bioactive molecules capable of rapid activation and thrombosis [48,49,50,51,52]. In Lippi’s meta-analysis, MPV was related to severe COVID-19 disease and was significantly increased in deceased patients [53]. Platelet over-activation due to impaired immune response, excessive cytokine release and dysregulation of ACE2 expression may contribute to the hypercoagulation that often occurs in COVID-19 disease [54]. Patients also express higher levels of P-selectin in activated or resting platelets, an increase in circulating platelet–leukocyte complexes, platelet accumulation and thrombin production [55,56,57]. Circulation of large, young platelets comes with a higher platelet activation potential and consequently a thrombogenic platelet potential [49,50]. This can be assessed with platelet volume indices (PVIs) related to platelet size, such as MPV, platelet distribution width (PDW) and platelet large cell ratio (P-LCR) [56]. Furthermore, higher MPV is associated with thrombocytopenia, often seen in COVID-19 patients [58]. It could be assumed that megakaryocytes increase the production of large young platelets, as a compensational mechanism to platelet consumption in pulmonary capillaries by microclot formation [59]. In the systematic review by Daniels et al., up to October 2021, 32 studies had examined a variety of markers related to platelet size (PVIs) in relation to the severity or mortality of COVID-19 disease [56]. They reported a general trend that higher marker values are related to disease severity and mortality, although longitudinal studies showed varying trends [56]. Researchers have recently adopted the MPV/PLT ratio as a more specific marker in the prognosis of platelet-related systemic thrombogenicity [60,61,62,63], while some have claimed superiority of the MPV/PLT ratio vs. MPV.

In the present study, we examine whether Hematology Laboratory can help to prompt screening and prognosis of COVID-19 hospitalized patients, by using the red cell anisocytosis index (RDW) and the kinetics–dynamics of platelets (platelet absolute number and platelet index MPV/PLT ratio as a measure of systemic thrombogenicity in COVID-19 disease), by recording and evaluating the above parameters at specific time points in the hospitalization in COVID-19 patients and in terms of mortality. This is the first study to investigate MPV/PLT ratio, platelets and RDW at multiple time-points during hospitalization.

## 2. Materials and Methods

### 2.1. Data Base Analysis

This study was retrospective and non-interventional, conducted at “Sismanoglio-A.Flemig Hospital/A.Flemig Hospital Unit”, Athens, Greece. Five hundred and eleven (511) hospitalized COVID-19 patients in the years 2020–2021 were included in this study, when the Hospital was designated by Ministerial decision as an “Exclusive Health Unit” for the care of COVID-19 patients, from 11 November 2020 to 8 June 2021 (the period that corresponded to the prevalence of the Delta subtype), according to the local epidemiological surveillance of the National Public Health Organization (https://eody.gov.gr/, (accessed on 31 December 2024)).

The Board of Directors and the Scientific Council of the General Hospital of Athens “Sismanoglio- A. Flemig” approved this study (Reference Number:16526/23-08-2023). This study was also registered at http://clinicaltrials.gov (accessed on 31 December 2024), code: NCT06755658.

The patients included in the present study were adults over 18 years of age, without myelodysplastic syndrome, iron deficiency or low B12 and folate values, who required hospital care for more than 24 h for COVID-19 infection, confirmed by molecular testing (rRT-PCR) and within one (1) month of positive diagnosis for COVID-19. The majority of the patients were admitted at the “Sismanoglio-A. Flemig Hospital”, while some were transferred from other Hospitals of the 1st Health Region of Attica where initial diagnosis had been made. Clinical and laboratory data were collected retrospectively from the patients’ files or from the laboratory information system (LIS) of the hospital. Study population characteristics are shown in Table 1.

The hematological laboratory values were performed using hematology analyzers subjected to internal and external quality control. In the General Hospital of Sismanoglio-A. Flemig, Marousi, Greece, Advia 2–120, XE-2100 Sysmex Automated Hematology Systems and Sysmex Ca-1500, Sysmex BCS XP Hemostasis Analyzers were used.

Patients were registered according to age, gender, comorbidities, maximum needs of FiO2, duration of hospitalization, COVID-19 disease severity, outcome (survival or death), RDW, platelets and MPV/PLT ratio values on admission, at discharge and mean values of hospitalization (Table 2, Table 3, Table A1 and Table A3). To follow the trend of the parameters, mean values of platelets and MPV/PLT ratio of the 1st and 2nd week of hospitalization, the nadir values of platelets, mean and nadir values of lymphocyte count (absolute number, percentage of nadir values and time point when the nadir value was observed), mean values and zenith of CRP and D-Dimers of all patients, as well as the score for DIC, based on ISTH criteria [65] for the deceased were recorded (Table 2, Table 3 and Table A1). Innovance D-Dimer assay was used for D-dimer testing, employing a cutoff of 0.50 mg/L FEU.

Time intervals of 1st and 2nd week of the parameters were chosen to be of interest as at approximately 7 to 14 days of disease COVID-19 onset, there is exacerbation of the clinical manifestations of the disease with a pronounced increase in inflammatory mediators and cytokines, when the virus starts a second attack [66].

Patients with increased RDW and thalassemia or thalassemia traits were included in this study without the RDW values except for RDW diff. The number of these patients are estimated to be up to 0.8% of the cohort of patients in this study.

The MPV/PLT ratio is defined and calculated as follows: MPV (fl) × 100

PLT (10^3^/μL)

Disease severity was defined as per the National Public Health Organization criteria (https://eody.gov.gr/) [67]. 1. Asymptomatic or presymptomatic disease: The patient is confirmed by molecular testing (rRT—PCR) for the SARS-CoV-2 virus but does not show clinical signs or symptoms. Laboratory and imaging tests (if possible) are not required. 2. Mild disease: The patient presents with fever, cough, symptoms of common cold, sore throat, loss of taste/smell, nausea/vomiting, diarrhea, fatigue. Oxygen saturation is >94% in room air and no shortness of breath is present. Laboratory and imaging tests (if possible) are not required. If a chest X-ray is performed, no findings are evident. 3. Moderate disease: The patient presents with symptoms of mild illness (fever, cough, sore throat, symptoms of common cold, loss of taste/smell, nausea/vomiting, diarrhea, fatigue) and has additional clinical signs of lower respiratory tract infection, but without shortness of breath and with oxygen saturation >94% in room air. Laboratory and imaging tests are required. If a chest X-ray is performed, few pulmonary infiltrates may appear. 4. Severe disease: The patient has oxygen saturation of <90% in room air or <94% but rapidly worsening, and/or signs of respiratory distress (30 breaths/minute, use of auxiliary respiratory muscles, inability to complete full sentence), and/or extensive infiltrates (>50%) in chest imaging and/or persistent high fever for more than 4–5 days or recurrence after days of apyrexia and/or combination of laboratory tests (lymphocytes < 1000/μL, ferritin > 1000 ng/mL, CRP > 100 mg/L with normal value < 5 mg/L) [67]. Critical disease: Patient presents with respiratory failure, septic shock and/or multiple organ dysfunction.

### 2.2. Statistical Analysis

For statistical analysis, the R programming language and Excel 2007 for Windows (Microsoft corporation) was used [68]. Statistical values were expressed for numerical variables as mean, standard deviation and median, [min, max] and for categorical variables as frequency of occurrence and percentages, %.

For data expressed in numerical value, the analysis was carried out using the *t*-test or the Mann–Whitney U test depending on data normality (as resulting from the Kolmogorov–Smirnov test). For qualitative data, the x^2^ test was used and when required, the Fisher exact test was applied.

In order to assess the relationship of various parameters (RDW, platelets, MVP/PLT ratio) in the prognosis of patients regarding their survival or disease severity, data were analyzed using the receiver operating characteristic (ROC) curves and the relevant area under the curve (AUC). Thresholds were selected so that the sensitivity value approaches that of the specificity (i.e., the difference between sensitivity and specificity was minimized). The level of statistical significance was set to <0.05 (*p* < 0.05) and when applicable tests were two-sided.

Furthermore, multivariable logistic regression analysis was performed for outcome and disease severity. Candidate variables for inclusion in the models were those found to be statistically significant (*p* < 0.05) in univariate analysis. Moreover, checks for high separability were also performed so that complete or quasi-complete separation could be avoided. Complete or quasi-complete separation occurs when one or more independent variables perfectly or nearly perfectly predict the outcome. This poses a problem as (a) the model fails to estimate coefficients accurately since estimates tend toward infinity, making the model unstable and the results unreliable; (b) *p*-values and confidence intervals may become undefined or extremely large; (c) the regression algorithm may fail to converge.

This is a retrospective study of exploratory nature, aiming at identifying potential associations between patient survival and disease severity with hematological indices and generating hypotheses rather than testing pre-specified ones. Thus, sample size was not calculated; instead, all available data were used. Furthermore, corrections for multiple comparisons are generally not recommended in exploratory analyses, as they may obscure potentially meaningful findings by increasing the risk of Type II errors [69,70]

The package pROC [71] was used for the analysis and production of ROC curves and the library caret [72] to identify variables with near zero variance [70]; furthermore, logistic regression was performed by the use of function glm (standard R package: stats, version 4.4.0).

## 3. Results

### 3.1. Outcome Analysis (Survival vs. Mortality)

Comparison for demographical data, comorbidities, disease severity, days in hospital and max FiO2% between the two groups of COVID-19 hospitalized patients showed (Table A1) that the deceased were older (mean age: 84.9 years ± 9.10 vs. 64.6 years ± 14.7, *p* < 0.001), with longer hospitalization (mean hospitalization time: 24.2 days ± 12.7 vs. 16.9 days ± 7.98, *p* < 0.001), with higher needs of oxygen (mean FiO2%: 76.3% ± 25.7 vs. 43.5% ± 18.6%, *p* < 0.001), were estimated with ECOG 4 at higher rates: 13.5% vs. 1.6%, *p* < 0.001) and with major comorbidities such as cardiovascular disease, diabetes mellitus, neoplastic disease, neurological disease (*p* < 0.001) and chronic renal failure (*p* < 0.00189). In addition, there were significant differences in gender and disease severity. A total of 22% of the deceased had a DIC(ISTH) score ≥ 5. As for disease outcome, our study showed that nearly all three parameters of RDW, platelets and MVP/PLT ratio, recorded at various time points differed significantly between survivors and deceased. Specifically for RDW, the mean value of hospitalization, admission, discharge and the difference from admission to discharge value were higher in the deceased. For platelets, the mean hospitalization, the 1st and 2nd week, the discharge and nadir values were significantly lower in the deceased. As for the MPV/PLT ratio, the mean value of the hospitalization, admission, 2nd week and discharge, zenith and nadir values, differed significantly with higher values in the deceased (Table 2).

**Figure 1:**
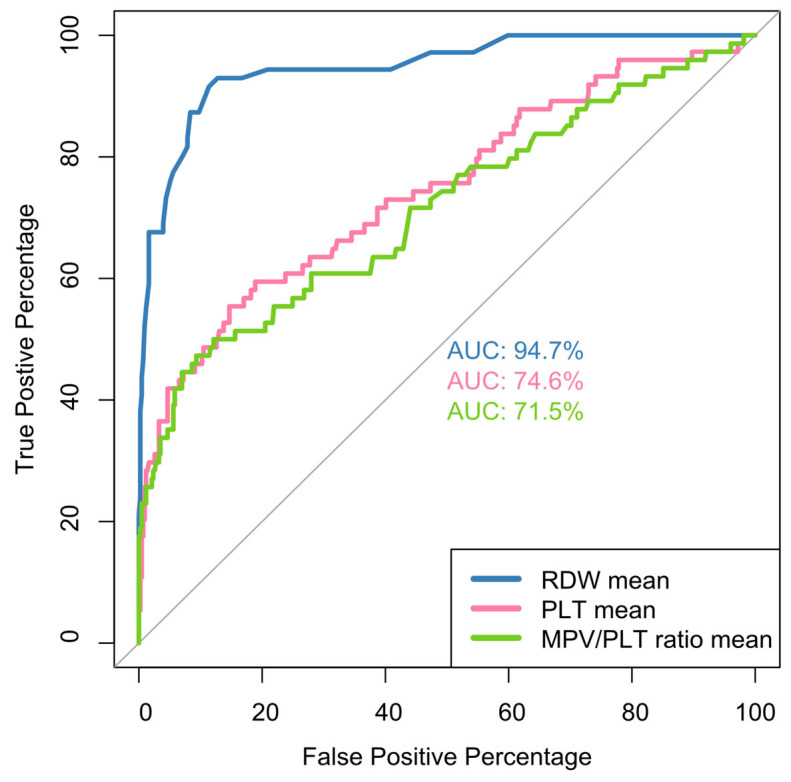
ROC curves for mean values RDW, PLTs, MPV/PLT ratio and COVID-19 disease outcome. For all cases in comparison, the comparison with the 50% diagonal line was *p* < 0.05.

**Figure 2:**
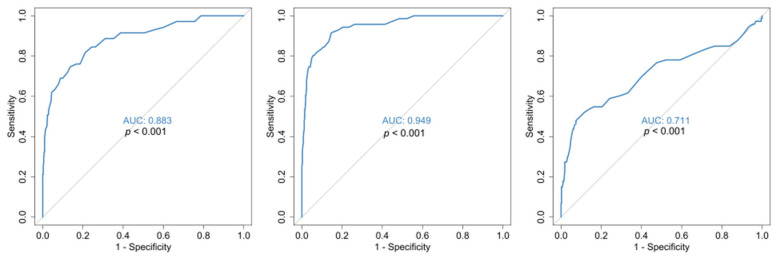
ROC curves for RDW on admission (**left**), RDW at discharge (**middle**) and RDW difference (**right**). All curves are for the prediction of COVID-19 disease outcome. *p*-values are for reference with the 50% diagonal line.

Median baseline RDW values were set at 13.4 [11.2, 17.2] vs. 15.4 [12.8, 22.3] for the deceased, *p* < 0.001 as shown in Table 2. As for the mortality, statistical analysis with ROC curves (Figure 1), showed that the mean hospitalization value RDW-CV is an excellent indicator for outcome prognosis (AUC = 0.947, with a cut-off value of 14.3% (sensitivity 91.5% and specificity 88.6%) for increased risk of mortality. In addition, in our study, admission value (baseline) RDW-CV has a threshold of >14.05% with AUC = 88.3% (sensitivity 81.7 and specificity 78.9) (Figure 2).

**Figure 3:**
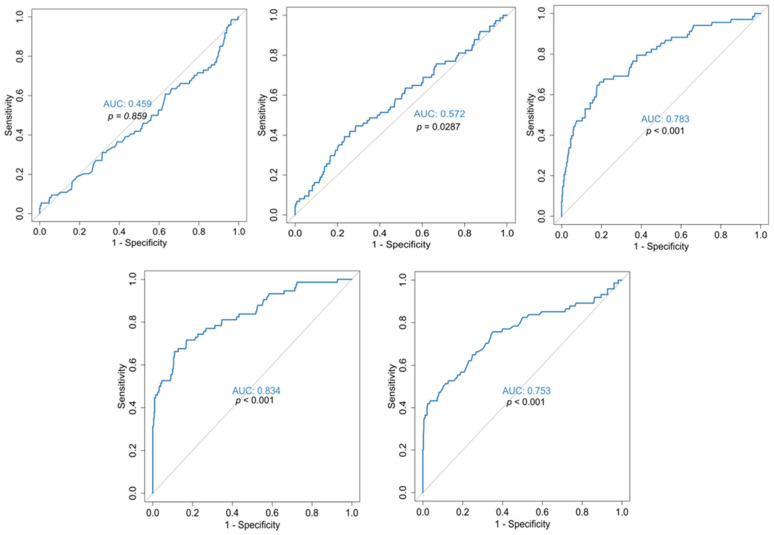
ROC curves for the prediction of COVID-19 outcome in relation to PLT on admission (**top left**), PLTs 1w (**top middle**), PLTs 2w (**top right**), PLTs at discharge (**bottom left**) and nadir PLTs (**bottom right**). *p*-values are for reference with the 50% diagonal line.

In the present study, in terms of COVID-19 mortality, mean hospitalization, 1st but mainly 2nd week, discharge and nadir values of platelets were significantly lower in the deceased (Table 2). However, statistical analysis with ROC curves showed that only the 2nd week platelet value is a satisfactory predictor of disease outcome (AUC = 78.3% sensitivity 69.1%, specificity 69.0%) with cut-off values < 279,250/μL (Figure 3). The nadir platelet value of survivors differed significantly from that in the deceased, with median values of 175,000 versus 119,000, respectively (Table 2).

**Figure 4:**
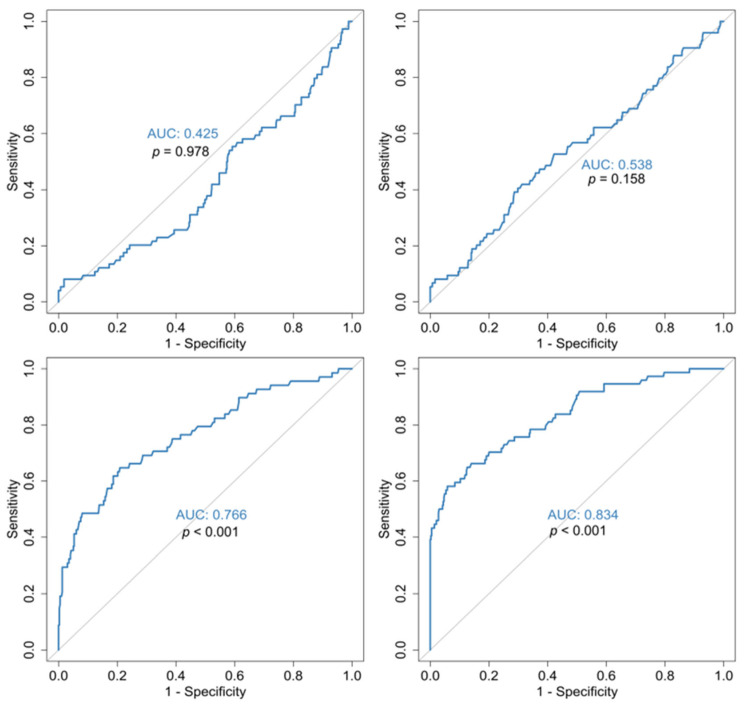
ROC curves for the prediction of COVID-19 outcome in relation to MPV/PLT ratio admission (**top left**), MPV/PLT ratio 1w (**top right**), MPV/PLT ratio 2w (**bottom left**), MPV/PLT ratio exit (**bottom right**). All *p*-values are in reference with the 50% diagonal line.

As for the outcome, mean hospitalization, admission, 2nd week and discharge values of MPV/PLT ratio were significantly higher in the deceased. Specifically, the 2nd week mean MPV/PLT ratio was 5.14 ± 3.48 in the deceased vs. 2.86 ± 1.18 in the survivors *p* < 0.001 (Table 2). ROC curves for the MPV/PLT ratio at various time points in this study identified that at admission and 1st week, it has no prognostic value, but the 2nd week MPV/PLT ratio has prognostic potential (AUC = 76.6%) with a threshold of >3.19 (sensitivity and specificity of 69.1% and 68.5%) (Figure 4).

**Figure A1:**
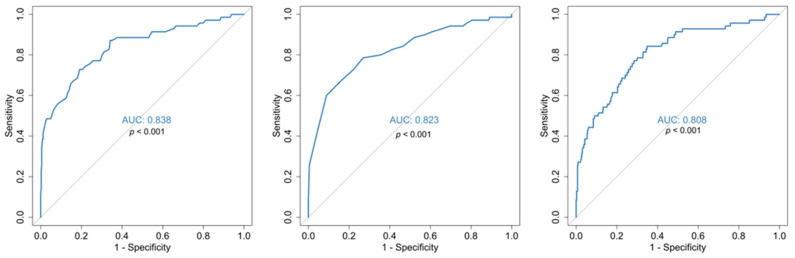
ROC curves for the prediction of COVID-19 outcome in relation to: LYMP # lowest (**left**), LYMP % lowest (**center**), LYMP mean (**right**). *p*-values are in reference with the 50% diagonal line.

**Figure A2:**
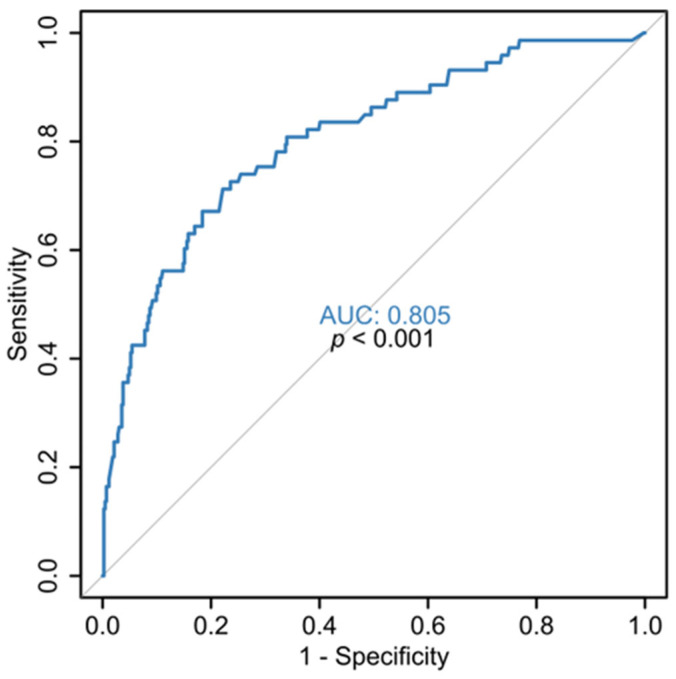
ROC curve for the capability of mean CRP to predict COVID-19 disease outcome. *p*-value is related to the 50% reference line.

**Figure A3:**
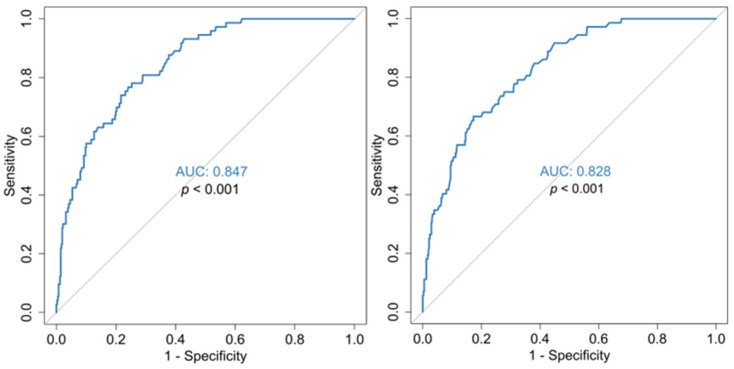
ROC curves for the predictive capability of DD highest (**left**) and DD mean (**right**) for COVID-19 disease outcome. *p*-values are in reference to the 50% diagonal line.

Additionally, concerning the outcome, the recorded values of absolute lymphocyte count (/μL) (mean hospitalization values and nadir) were significantly lower, the D-Dimer(mg/L), and CRP (mg/L) values (mean hospitalization values, and zenith) were significantly higher in the deceased. It is also noted that the deceased had a much lower nadir lymphocytes percentage, and that the nadir of lymphocytes differed significantly between the two groups, in all weeks of hospitalization. It is worth noting that the lymphocyte nadir of the survivors occurred in the 1st week mainly, as well as the zenith of CRP values (Table 2). ROC curves showed that these parameters can be of prognostic value (AUC ≥ 0.80 sensitivity and specificity ≥72.75%) (Figure A1, Figure A2 and Figure A3). Cut-off values of lymphocytes, DD and CRP are shown in Table A2.

### 3.2. Disease Severity Analysis (Mild/Moderate vs. Severe/Critical)

Comparison between the two groups of COVID-19 hospitalized patients, mild/moderate vs. severe/critical, showed (Table A3) that the median age was 65.5 [37.0, 95.0] vs. 68.0 [22.0, 99.0], respectively. The two groups did not differ significantly for age, duration of hospitalization, ECOG 4 or for the presence of comorbidities except for arterial hypertension, neurological and psychiatric disease. COVID-19 hospitalized patients with mild/moderate disease, who were on antidepressants, dementia or psychiatric treatment, mostly came from care units, or nursing homes and stayed in hospital till they became PCR negative. Patients with severe/critical disease were mostly males, had higher needs of oxygen (Mean FiO2 50.8% vs. 22.3%) and were estimated with a fatal outcome to 15.6%

**Figure 5:**
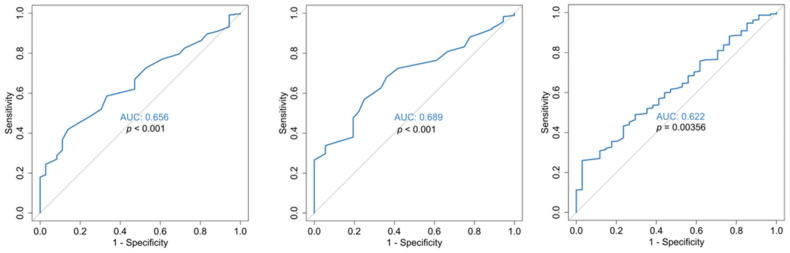
ROC curves for the predictive capability of RDW on admission (**left**), RDW mean (**middle**), PLTs on admission (**right**) for disease severity. *p*-values are in reference to the 50% diagonal line.

As for disease severity (Table 3), it appears that admission and hospitalization mean RDW-CV, although significantly different between mild/moderate and severe/critical groups, is not of prognostic value (AUC 0.65–0.68, Figure 5).

Regarding disease severity (Table 3), the platelet value did not differ as a mean hospitalization value, or as values of the 1st, 2nd week of hospitalization and discharge, in contrast to the admission and nadir values. Its predictive value was not confirmed by ROC curves (Figure 5), and therefore it does not seem to help in the assessment of the disease severity.

**Figure 6:**
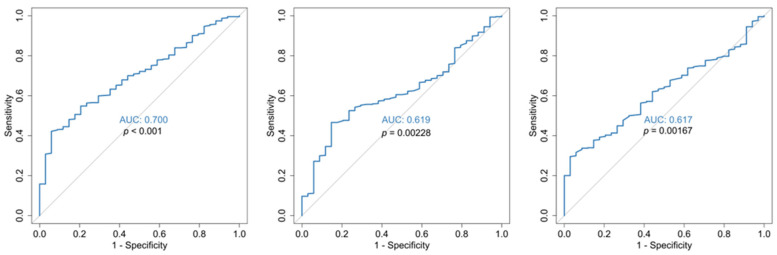
ROC curves for disease severity prediction of admission MPV/PLT ratio (**left**), MPV/PLT ratio 1w (**center**) and mean value of MPV/PLT ratio (**right**). *p*-values are in reference to the 50% diagonal line.

Admission MPV/PLT ratio (Table 3) was significantly higher in patients with severe/critical disease compared to those with mild/moderate disease–mean value 5.75 ± 2.66 vs. 4.14 ± 1.44 and median value 5.49 [1.21, 27.1] vs. 4.18 [1.57, 7.70], *p* < 0.001, respectively. Mean hospitalization MPV/PLT ratio values, at 1st week and zenith, were also significantly different. In respect of disease severity (mild/moderate vs. severe/critical), further statistical analysis showed that only the admission MPV/PLT ratio can be used to a moderate level (AUC 0.70) as a prognostic marker (Figure 6).

### 3.3. Multivariable Analysis

Multivariable logistic regression analysis for survival was also performed. Variables for inclusion in the model were those hematological parameters found to be statistically significant in the univariate analysis (Table 2) and demographics such as age and gender. Nevertheless, many variables were not included in the logistic regression model due to high discriminatory power leading to (quasi-)complete separation (q.v., statistical analysis).

Excluded variables were (see Table 2 for more details) for possible separation: admission MPV/PLT ratio, admission RDW, discharge RDW, RDW diff, discharge PLTs, PLT nadir, discharge MPV/PLT ratio, DD highest, DD mean, LYMP # lowest, LYMP# lowest when, LYMP % lowest, LYMP mean, CPR highest at, MPV/PLT highest and MPV/PLT lowest. Eventually, the key variables included to ensure model convergence for survival are those defined in Table 4. The results indicated that increased age and elevated RDW mean value are associated with a higher mortality risk and in terms of outcome, only age and RDW mean (*p* < 0.0001 OR 5.8844) are independent variables.

In multivariable logistic regression analysis for disease severity, variables for inclusion in the model were those found to be statistically significant in univariate analysis (Table 3) and demographics such as age and gender. However, during the screening process, several variables were excluded due to their high discriminatory power, specifically, discharge RDW, PLTs nadir, MPV/PLT ratio 1w, DD highest, DD mean, mean LYMP, LYMP # lowest, LYMP % lowest, CRP highest, CPR highest at, CRP mean, MPV/PLT highest and MPV/PLT lowest. Ultimately, only a subset of key variables was retained in the model: age, gender, mean RDW, mean MPV/PLT ratio, admission RDW, admission MPV/PLT ratio and admission PLTs (Table 5). Model convergence was finally confirmed. The results showed that female patients had better odds for mild/moderate disease. Higher values of mean RDW and admission MPV/PLT ratio were associated with severe/critical illness, and in terms of disease severity are, along with gender, the only independent variables.

## 4. Discussion

The novelty in our study is the investigation of the MPV/PLT ratio as a marker of platelet-related thrombogenity, not merely on admission but at several time points during hospitalization, thereby investigating its use for prognosis of hospitalized COVID-19 patients in conjunction with the RDW and platelet absolute number indexes.

Several authors reported on the association of baseline values of RDW with COVID-19. As RDW emerges as an indicator with consistency in COVID-19 prognosis, we thought that we should incorporate it in a prognostic model that a hematology lab could offer, compare our results with the corresponding ones in the literature and define the cut-off values.

The increase in RDW, i.e., RBC anisocytosis, reflects a dysfunction of erythrocyte homeostasis that involves a disturbed erythropoiesis and abnormal metabolism or survival. Foy reported, as demonstrated by semi-mechanistic mathematical models on the dynamics of in vivo RBC populations, that under certain conditions, the body can delay the clearance of aged red blood cells from the circulation [39,73]. RBCs, as they age, become smaller in size and lose Hb [74]. A slight slowdown in the rate of their removal from the circulation results in populations of RBCs with smaller size, increased anisocytosis and consequently raised RDW. This slowdown in clearance coincides with and comes as a compensation for the body’s effort to maintain a stable mass of RBCs in disorders where a decrease in their production occurs [74]. Inflammation, which is common in many pathological disorders, may be a mechanism responsible for anisocytosis, since many pro-inflammatory cytokines inhibit the synthesis or the response to erythropoietin and consequently the production of red blood cells [75]. In addition to inflammation, various etiologies have been proposed in the literature, such as shortening of telomere length, oxidative stress, red cell fragmentation, dyslipidemia, malnutrition, hypertension and abnormality of erythropoietin activity [3].

A specific mechanism linking erythrocyte anisocytosis to COVID-19 disease has not been defined. If RDW is merely a marker of disease in general, it may not be specifically associated with COVID-19 disease [39]. Since the increase in RDW is associated with conditions that disrupt erythropoiesis, the overproduction of inflammatory cytokines in severe COVID-19 disease, by reduced production or unresponsiveness to erythropoietin, could be a mechanism for that [75,76]. In severe COVID-19 disease, sepsis-induced coagulopathy, disseminated intravascular coagulation (DIC) as well as thrombotic microangiopathy can lead to erythrocyte damage, morphological abnormalities, fragmentation of red blood cells and consequently to an increase in RDW [77]. Gavrilaki et al. reported that the symptoms of severe COVID-19 disease are similar in the phenotype and pathophysiology of complement-induced thrombotic micro-angiopathies (TMAs) [77]. Bone marrow direct injury due to SARS-CoV-2 infection and hence to disturbance of red cell homeostasis may be another mechanism, such as hemophagocytosis [78,79]. Other suggested mechanisms could be the structural changes that SARS-CoV-2 infection induces to the lipids and proteins of the membrane of circulating RBCs by oxidative damage [80,81] or the autoimmune hemolytic anemia—AIHA associated with COVID-19 disease reported in the literature [78,82]

Our results suggest that an elevated RDW on admission is associated with a high mortality risk for COVID-19 patients, in line with reports from other investigators [39,40,41,42,44] and with Foy’s results as well, in their multicenter retrospective study [39] providing baseline values of the RDW. Further statistical analysis with ROC curves (Figure 1), showed that the mean hospitalization and the baseline admission RDW-CV values are excellent indicators for outcome prognosis (Figure 2). However, our value is lower than the one defined in Foy’s study (>14.5%). These differences might be explained by the fact that their data were extracted exclusively from one type of hematology analyzer (Sysmex); meanwhile, in our study, data were obtained from at least three types of hematology analyzers of known manufacturing companies (Advia 2120, DXH800 Coulter, XE2100 Sysmex) used by the hospitals of the 1st Health Region of Attica; so, a degree of bias [83] cannot be avoided in relation to the RDW value. It could also be attributed to the early lockdown implementation in our country, and the meticulous application of preventive measures in the first wave of the pandemic, as Karampitsakos et al. [40] commented in their study.

As for COVID-19 disease severity, it appears that the admission and hospitalization mean RDW-CV values in our study (Table 3), although with a significant difference between the mild/moderate and severe/critical groups, do not offer prognosis in terms of disease severity unlike the findings of Atik et al. [84]. This might be due to the fact that in our study, both groups involved patients of older age (median age > 65) in contrast to Atik’s groups (median age of mild/moderate: 57–60). Foy reports that the risk for COVID-19 mortality from elevated RDW is lower at ages ≥ 70 compared to younger ages [39].

Regarding platelets in terms of outcome (COVID-19 mortality), although mean hospitalization, 1st but mainly 2nd week, discharge and nadir platelet values were significantly lower in the deceased (Table 2), only the 2nd week platelet value was a satisfactory predictor for disease outcome, as shown by statistical analysis with ROC curves. The fact that the 2nd week platelet value can be of prognostic significance is supported by Zhao et al.’s study [85], where the early reduction in platelets during hospitalization was associated with mortality in COVID-19 patients, as platelet values of the deceased were progressively decreasing one week after their admission. Comparing our results with that of other studies, mean platelet values in the survivors differed significantly from the deceased, as in Ruan et al.’s study [86], although our values were higher. The nadir platelets of survivors in our study differed significantly from those of the deceased, as in Yang et al.’s retrospective study [87] of 1476 patients, although their values of the deceased were, to some degree, lower. Possibly, our higher values were partly due to persistent care and support of patients regardless of age.

As for COVID-19 disease severity, the platelet parameter did not differ either as a mean hospitalization value, or during the 1st and 2nd weeks of hospitalization or at discharge contrary to the admission and nadir value, but its predictive value was not confirmed by ROC curves and therefore does not seem to help with the disease severity estimation, in line with the conclusions of the study of Wang C et al. [88] and of Atik [84].

Admission MPV/PLT ratio was significantly higher in COVID-19 patients with severe/critical disease compared to those with mild/moderate disease. Our findings are in line with reports by Atik et al., although their values are higher [84]. Similarly, in Zhong et al.’s study, admission MPV/PLT ratio was associated with severe pneumonia of COVID-19 to a statistically significant degree [89]. Their higher values may have been due to the fact that their data came from a single center and were provided exclusively from one type of hematology analyzer (Sysmex in the Atik’s study), in contrast to our data that came, as mentioned previously, from hematology analyzers with different technologies. In Lippi et al.’s study, although there is an optimal degree of analytical quality and comparability for PLT values, among different types of hematology analyzers of the known manufacturing companies, it is discussed that the bias in MPV can be quite large [90].

A matter of interest in our study is the MPV/PLT ratio at various time points as for outcome and disease severity. Specifically, as for outcome, mean hospitalization, admission, 2nd week and discharge MPV/PLT ratio, these differed significantly, with higher values in the deceased. Further statistical analysis by ROC curves pointed out that the MPV/PLT ratio on admission and at 1st week has no prognostic value, but the 2nd week MPV/PLT ratio value has prognostic potential. Furthermore, with regard to disease severity at various time points in this study, only the admission MPV/PLT ratio has a prognostic value to a moderate degree. The above findings could offer an alarm signal for persistent and aggressive therapeutic care implementation.

Concerning the outcome of COVID-19 disease in our study, absolute lymphocyte count values, D-Dimer and CRP significantly differed between the two COVID-19 patients’ groups. In the literature, lymphopenia is a prominent finding in severe cases of COVID-19 disease [91,92,93,94], and the sequential assessment of lymphocyte dynamics can be predictive for the patient’s outcome [93]. The value of DD is indicative of endovascular thrombosis and is reported as an independent mortality risk predictor of COVID-19 disease [95]. CRP, an indicator of inflammation, is particularly elevated in cytokine storm that can occur in COVID-19 patients and is associated with disease mortality [96]. Statistical analysis with ROC curves showed that these parameters can also be of prognostic value to a high degree, with the potential to add to a possible prognostic system offered by the hematology laboratory.

Multivariable analysis using logistic regression for survival showed that increased age and an elevated RDW mean value are associated with higher mortality risk, and in terms of outcome, only age and RDW mean are independent variables. Nevertheless, it must be taken into account that due to high separability issues, several parameters at various time points were excluded from the model, such as the RDW baseline. Concerning COVID-19 severity, multivariable logistic regression analysis indicated the MPV/PLT ratio on admission as an independent variable, in addition to the RDW mean value and gender. Female patients had better odds for mild/moderate disease. Higher values of RDW mean and MPV/PLT ratio on admission were associated with severe/critical illness.

Finally, it is worth noting that the values of the RDW and MPV parameters show a degree of bias depending on the technology of the hematology analyzer each laboratory employs, in contrary to classic parameters such as Hb, MCV or platelets. For the results to be comparable, it is necessary to ensure that the manufacturing companies comply with the recommendations of the International Council for Standardization in Haematology (ICSH) [3,83,90,97].

As per the above, lack of harmonization could have been a limitation in our study. Considering the bias due to manufacturing technology for RDW and MPV, in order to elucidate more clearly the trend of these parameters as well as their prognostic significance, it would have been useful if a hematology system analyzer of one type of technology had been used. Our study is from a single center, with admission of patients from secondary sources and this is also a limitation. Moreover, the time of enrollment in this study coincided with the pre- and initial vaccination period and there were no complete data on the vaccination status of the patients except in isolated cases. That might affect the generalization of results in later periods of the pandemic. This study is also retrospective, so well designed, prospective studies are needed to confirm the certainty of results.

## 5. Conclusions

In our study, the erythrocyte and platelet indices such as RDW, platelet absolute numbers and MPV/PLT ratio at specific time points in COVID-19 hospitalized patients, were significantly associated with outcome and disease severity, but their predictive power, based on ROC curves statistical analysis, ranged from excellent to satisfactory or were of no discriminative value, depending on the hospitalization timeline. Along with other parameters that a routine hematology laboratory may provide in a short time, lymphocyte count and DD ± CRP could be of predictive value. In our prognostic model based on multivariable logistic regression, only RDW mean value for mortality, and admission MPV/PLT ratio with RDW mean for severity, emerged as independent variables and may aid risk stratification. As for RDW and MPV parameters, the lack of harmonization could be an obstacle to their use as predictive indicators on a large scale.

## Figures and Tables

**Table 1:** Demographic characteristics, disease severity, performance status, needs of FiO2 (%), duration of hospitalization, antiviral/immunomodulatory therapy and outcome for the study population.

Characteristics	N, %
Age	
Mean (SD)	67.7 (15.7)
Median [Min, Max]	68.0 [22.0–99.0]
Gender	
Male	264 (51.7%)
Female	247 (48.3%)
Other Nationalities	38 (7.4%)
Severity	
Mild	2 (0.4%)
Moderate	34 (6.7%)
Critical	19 (3.7%)
Severe	456 (89.2%)
2-Level Severity	
Mild/Moderate	36 (7.0%)
Severe/Critical	475 (93.0%)
ECOG 4	
Yes	17 (3.3%)
No	494 (96.7%)
FiO2	
Mean (SD)	48.7% (0.232)
Median [Min, Max]	40% [21%, 100%]
Missing	5 (1.0%)
Days in hospital	
Mean (SD)	17.9 (9.15)
Median [Min, Max]	15.0 [5.00, 72.0]
No Remdesivir	154 (30.1%)
R	346 (67.7%)
R + A	5 (1.0%)
R + T	3 (0.6%)
R + B	1 (0.2%)
T	1 (0.2%)
A	1 (0.2%)
Outcome	
Improvement	426 (83.4%)
Death	74 (14.5%)
Discharge with O_2_ support	6 (1.2%)
Missing	5 (1.0%)

ECOG: performance status scoring; FiO2%: maximum fraction of inspired oxygen needed; A: anakinra; T: tocilizumab; Β: baricitinib; R: remdesivir. All patients received standard care as defined by national protocols [64]. Most patients with severe disease received, in addition to standard prophylaxis with low molecular weight heparin, antibiotic treatment with azithromycin-ceftriaxone and dexamethasone. The administration of remdesivir alone or in co-administration with interleukin inhibitors (tocilizumab and anakinra) or JAK inhibitors (baricitinib) is shown in Table 1.

**Table 2:** COVID-19 disease mortality in relation to hematological characteristics of hospitalized patients.

Characteristics	Death (N = 74)	Survival (N = 432)	*p*-Value
RDW mean			
Mean (SD)	16.3 (1.98)	13.4 (0.805)	<0.001
Median (min, max)	16.0 [13.2, 23.4]	13.3 [11.4, 17.3]	
PLT mean			
Mean (SD)	207,000 (87,400)	281,000 (83,500)	<0.001
Median (min, max)	189,000 [23,500, 490,000]	269,000 [102,000, 616,000]	
MPV/PLT ratio mean			
Mean (SD)	7.80 (9.60)	3.99 (1.39)	<0.001
Median (min, max)	5.42 [1.81, 73.1]	3.85 [1.29, 9.60]	
Admission RDW			
Mean (SD)	15.8 (2.15)	13.4 (0.876)	<0.001
Median (min, max)	15.4 [12.8, 22.3]	13.4 [11.2, 17.2]	
Admission MPV/PLT ratio			
Mean (SD)	5.65 (4.15)	5.63 (2.28)	0.0392
Median (min, max)	4.89 [1.81, 27.1]	5.46 [1.21, 15.3]	
Admission PLTs			
Mean (SD)	220,000 (89,400)	209,000 (82,200)	0.258
Median (min, max)	201,000 [43,000, 504,000]	185,000 [66,000, 683,000]	
Discharge RDW			
Mean (SD)	17.0 (2.11)	13.6 (0.984)	<0.001
Median (min, max)	16.7 [13.3, 24.0]	13.4 [11.5, 18.0]	
RDW dif			
Mean (SD)	1.23 (1.75)	0.163 (0.755)	<0.001
Median (min, max)	1.00 [−3.80, 6.80]	0.100 [−2.10, 4.00	
Discharge PLTs			
Mean (SD)	166,000 (110,000)	322,000 (118,000)	<0.001
Median (min, max)	150,000 [9000, 508,000]	303,000 [93,000, 744,000]	
Discharge MPV/PLT ratio			
Mean (SD)	14.8 (23.2)	3.01 (1.42)	<0.001
Median (min, max)	6.56 [1.61, 128]	2.66 [0.850, 8.40]	
PLTs 1w			
Mean (SD)	223,000 (91,800)	245,000 (89,200)	0.0467
Median (min, max)	206,000 [23,500, 493,000]	222,000 [93,300, 616,000]	
MPV/PLT ratio 1w	4.90 [1.83, 73.1]	4.50 [1.29, 11.8]	0.3
PLT 2w			
Mean (SD)	234,000 (105,000)	347,000 (113,000)	<0.001
Median (min, max)	220,000 [43,000, 570,000]	337,000 [118,000, 744,000]	
MPV/PLT ratio 2w			
Mean (SD)	5.14 (3.48)	2.86 (1.18)	<0.001
Median (min, max)	4.16 [1.36, 20.1]	2.60 [0.850, 8.40]	
PLTs nadir	119,000 [9000, 367,000]	175,000 [66,000, 605,000]	<0.001
MPV/PLT highest			
Mean (SD)	16.6 (23.2)	5.97 (2.24)	<0.001
Median (min, max)	8.18 [2.67, 128]	5.70 [1.49, 15.1]	
MPV/PLT lowest			
Mean (SD)	3.85 (3.66)	2.41 (0.969)	<0.001
Median (min, max)	3.16 [1.09, 27.1]	2.19 [0.850, 6.69]	
DD highest			
Median (min, max)	4.69 [0.790, 99.0]	1.02 [0.190, 45.4]	<0.001
Mean DD			<0.001
Mean	4.04(6.02)	1.07(1.28)	
Median (min, max)	2.32 [0.510, 33.8]	0.720 [0.190, 14.2]	
Mean LYMP			
Mean (SD)	747 (341)	1180 (412)	<0.001
Median (min, max)	694 [246, 1860]	1130 [361, 2620]	
LYMP # lowest			
Mean (SD)	404 (241)	749 (313)	<0.001
Median (min, max)	370 [80.0, 1280	700 [190, 2210]	
LYMP % lowest			
Median (min, max)	0.0400 [0.0100, 0.630]	0.110 [0.0200, 0.360]	<0.001
LYMP # lowest when			
1w	27 (36.5%)	341 (78.9%)	<0.001 fx
2w	16 (21.6%)	63 (14.6%)	
3w	12 (16.2%)	17 (3.9%)	
4w	10 (13.5%)	4 (0.9%)	
5w	4 (5.4%)	1 (0.2%)	
8w	1 (1.4%)	0 (0%)	
6w	0 (0%)	1 (0.2%)	
Mean CRP	71.0 (36.0)	33.5 (29.4)	<0.001
CRP highest	152 (78.0)	82.7(67.4)	<0.001
CPR highest at:			
1w	29 (39.2%)	369 (85.4%)	<0.001 fx
2w	9 (12.2%)	38 (8.8%)	
3w	14 (18.9%)	10 (2.3%)	
4w	11 (14.9%)	3 (0.7%)	
5w	6 (8.1%)	2 (0.5%)	
6w	2 (2.7%)	0 (0%)	
7w	2 (2.7%)	0 (0%)	

Statistical analysis: Mann–Whitney U, unless fx. fx: Fisher exact test. Min and max value in square brackets. RDW mean: mean hospitalization RDW-CV(%); Admission RDW: RDW-CV(%) baseline; Discharge RDW: RDW-CV(%) at discharge; RDW diff: difference in values RDW admission–discharge; PLT mean: mean hospitalization platelets(/μL); Admission PLTs: platelet (/μL) baseline; Discharge PLTs: platelets (/μL) at discharge; PLTs 1w: mean 1st week platelets (/μL); PLTs 2w: mean 2nd week platelets (/μL); PLTs nadir (/μL): lowest platelets; MPV/PLT ratio mean: mean hospitalization MPV/PLT ratio; Admission MPV/PLT ratio; MPV/PLT ratio 1w; MPV/PLT ratio 2w; Discharge MPV/PLT ratio: MPV/PLT ratio at discharge; MPV/PLT ratio highest/lowest: MPV/PLT ratio zenith/nadir; Mean DD: mean hospitalization DD((mg/L); DD highest: zenith value DD (mg/L); Mean LYMP: mean hospitalization absolute lymphocyte number (/μL); LYMP # Lowest: nadir absolute lymphocyte number (/μL); LYMP % Lowest: % of nadir lymphocytes; LYMP when: week of nadir lymphocytes; Mean CRP: mean hospitalization CRP (mg/L); CRP highest: zenith value CRP (mg/L); CPR highest at: week of zenith CRP value. Missing values of RDW, PLTs, MPV/PLT ratio parameters ranged up to 0.6–0.8%. Missing values of LYMP, CRP parameters estimated up to 2% and DD to 4.5%.

**Table 3:** Hematological characteristics according to disease severity.

Characteristics	Mild/Moderate (N = 36)	Severe/Critical (N = 475)	*p*-Value
RDW mean			
Mean (SD)	13.1 (0.570)	13.9 (1.51)	<0.001
Median [Min, Max]	13.0 [11.9, 14.1]	13.5 [11.4, 23.4]	
PLTs mean			
Mean (SD)	271,000 (73,300)	269,000 (89,200)	0.774
Median [Min, Max]	266,000 [169,000, 499,000]	261,000 [23,500, 616,000]	
Admission MPV/PLT ratio			
Mean (SD)	4.14 (1.44)	5.75 (2.66)	<0.001
Median [Min, Max]	4.18 [1.57, 7.70]	5.49 [1.21, 27.1]	
MPV/PLT ratio mean			
Mean (SD)	3.55 (0.889)	4.63 (4.22)	0.0228
Median [Min, Max]	3.49 [1.55, 5.46]	3.98 [1.29, 73.1]	
Admission RDW			
Mean (SD)	13.1 (0.687)	13.8 (1.46)	0.00175
Median [Min, Max]	13.1 [11.4, 14.7]	13.5 [11.2, 22.3]	
Discharge RDW			
Mean (SD)	13.2 (0.604)	14.1 (1.74)	<0.001
Median [Min, Max]	13.1 [12.3, 14.4]	13.7 [11.5, 24.0]	
RDW diff			
Mean (SD)	0.0556 (0.466)	0.336 (1.06)	0.183
Median [Min, Max]	0 [−0.800, 0.900]	0.200 [−3.80, 6.80]	
Admission PLTs			
Mean (SD)	244,000 (96,900)	208,000 (81,700)	0.0175
Median [Min, Max]	211,000 [131,000, 497,000]	185,000 [43,000, 683,000]	
PLTs 1w			
Mean (SD)	245,000 (85,800)	240,000 (90,100)	0.774
Median [Min, Max]	221,000 [131,000, 499,000]	221,000 [23,500, 616,000]	
PLTs 2w			
Mean (SD)	300,000 (95,100)	331,000 (120,000)	0.185
Median [Min, Max]	301,000 [156,000, 523,000]	326,000 [43,000, 744,000]	
Discharge PLTs			
Mean (SD)	299,000 (99,800)	298,000 (131,000)	0.887
Median [Min, Max]	294,000 [151,000, 610,000]	293,000 [9000, 744,000]	
PLT nadir			
Mean (SD)	206,000 (71,900)	176,000 (71,000)	0.0112
Median [Min, Max]	186,000 [120,000, 490,000]	169,000 [9000, 605,000]	
MPV/PLT ratio 1w			
Mean (SD)	4.05 (1.33)	5.05 (3.86)	0.0207
Median [Min, Max]	4.08 [1.55, 7.70]	4.70 [1.29, 73.1]	
MPV/PLT ratio 2w			
Mean (SD)	3.15 (1.16)	3.22 (1.96)	0.51
Median [Min, Max]	2.86 [1.31, 5.38]	2.73 [0.850, 20.1]	
Discharge MPV/PLT ratio			
Mean (SD)	3.02 (1.12)	4.88 (10.2)	0.616
Median [Min, Max]	2.69 [1.25, 5.38]	2.91 [0.850, 128]	
Missing	2 (5.6%)	1 (0.2%)	
DD highest			
Mean (SD)	1.29 (1.19)	3.66 (8.66)	0.0113
Median [Min, Max]	0.830 [0.190, 5.70]	1.24 [0.190, 99.0]	
Missing	1 (2.8%)	22 (4.6%)	
Mean DD			
Mean (SD)	0.811 (0.546)	1.57 (2.89)	0.0305
Median [Min, Max]	0.710 [0.190, 2.15]	0.840 [0.190, 33.8]	
Missing	1 (2.8%)	24 (5.1%)	
LYMP # Lowest			
Mean (SD)	1060 (348)	673 (310)	<0.001
Median [Min, Max]	1010 [520, 2210]	620 [80.0, 1890]	
LYMP # lowest at:			
1w	25 (69.4%)	345 (72.6%)	0.203 fx
2 w	7 (19.4%)	72 (15.2%)	
4 w	3 (8.3%)	11 (2.3%)	
3 w	0 (0%)	31 (6.5%)	
5 w	0 (0%)	5 (1.1%)	
6 w	0 (0%)	1 (0.2%)	
8 w	0 (0%)	1 (0.2%)	
LYMP % lowest			
Mean (SD)	0.245 (0.0742)	0.110 (0.0737)	<0.001
Median [Min, Max]	0.250 [0.110, 0.360]	0.0900 [0.0100, 0.630]	
Mean LYMP			
Mean (SD)	1400 (441)	1100 (423)	<0.001
Median [Min, Max]	1350 [717, 2620]	1050 [246, 2580]	
CRP highest			
Mean (SD)	27.4 (24.3)	98.1 (73.5)	<0.001
Median [Min, Max]	22.2 [2.00, 103]	76.9 [2.00, 440]	
CPR highest at:			
1 w	27 (75.0%)	373 (78.5%)	0.61 fx
2 w	5 (13.9%)	43 (9.1%)	
3 w	0 (0%)	26 (5.5%)	
4 w	0 (0%)	14 (2.9%)	
5 w	0 (0%)	8 (1.7%)	
6 w	0 (0%)	2 (0.4%)	
7 w	0 (0%)	2 (0.4%)	
Mean CRP			
Mean (SD)	13.7 (13.5)	41.1 (29.9)	<0.001
Median [Min, Max]	11.0 [2.00, 70.0]	33.6 [1.24, 162]	
MPV/PLT highest			
Mean (SD)	4.72 (1.38)	7.75 (10.1)	<0.001
Median [Min, Max]	4.57 [1.75, 8.55]	6.14 [1.49, 128]	
MPV/PLT lowest			
Mean (SD)	2.48 (0.854)	2.64 (1.78)	0.905
Median [Min, Max]	2.32 [1.25, 4.21]	2.30 [0.850, 27.1]	

fx: Fisher exact test, otherwise Mann–Whitney U is used. RDW mean: mean hospitalization RDW-CV(%); Admission RDW: RDW-CV(%) baseline; Discharge RDW: RDW-CV(%) at discharge; RDW diff: difference in values RDW admission–discharge; PLTs mean: mean hospitalization platelets (/μL); Admission PLTs: platelets (/μL) baseline; Discharge PLTs: platelets(/μL) at discharge; PLTs 1w: mean 1st week platelets (/μL); PLTs 2w: mean 2nd week platelets (/μL); PLTs nadir (/μL): lowest platelets; MPV/PLT ratio mean: mean hospitalization MPV/PLT ratio; Admission MPV/PLT ratio, MPV/PLT ratio 1w, MPV/PLT ratio 2w, Discharge MPV/PLT ratio: MPV/PLT ratio at discharge, MPV/PLT ratio highest/lowest: MPV/PLT ratio zenith/nadir; Mean DD: mean hospitalization DD ((mg/L); DD highest: zenith value DD (mg/L); Mean LYMP: mean absolute lymphocyte number (/μL) hospitalization; LYMP # Lowest: nadir absolute lymphocyte number (/μL); LYMP % Lowest: % of nadir lymphocytes; LYMP when: week of nadir lymphocytes; Mean CRP: mean CRP hospitalization (mg/L); CRP highest: zenith value CRP (mg/L); CPR highest at: week of zenith CRP value. Missing values of RDW, PLTs, MPV/PLT ratio parameters ranged up to 0.6–0.8%. Missing values of LYMP, CRP parameters estimated up to 2% and DD up to 4.6–5.1%.

**Table 4:** Multivariable logistic regression for mortality risk.

Characteristic	Estimate	Std Error	*p*-Value	OR (95% CI)
Age	0.0672	0.0263	0.0107	1.0696 (1.0157–1.1262)
Gender Female	0.0844	0.4971	0.8652	1.088 (0.4107–2.8827)
RDW mean	1.7723	0.2668	<0.0001	5.8844 (3.4884–9.9263)
PLT mean	0.0000	0.0000	0.9143	1 (1–1)
MPV/PLT ratio mean	0.3873	0.2926	0.1856	1.473 (0.8301–2.6138)
PLT 2w	−0.000001	0.000007	0.8261	1 (1–1)
MPV/PLT ratio 2w	0.0534	0.2619	0.8384	1.0549 (0.6314–1.7623)

**Table 5:** Multivariable logistic regression for risk of severe/critical illness.

	Estimate	Std Error	*p*-Value	OR (95% CI)
Age	−0.0226	0.0149	0.1276	0.9776 (0.9495–1.0065)
Gender Female	−1.5338	0.4468	0.0006	0.2157 (0.0899–0.5178)
RDW mean	1.6300	0.5256	0.0019	5.1039 (1.8219–14.298)
Admission MPV/PLT ratio	0.8461	0.2352	0.0003	2.3306 (1.4698–3.6956)
MPV/PLT ratio mean	−0.1815	0.1083	0.0939	0.834 (0.6745–1.0313)
Admission RDW	−0.2380	0.4427	0.5908	0.7882 (0.331–1.8769)
Admission PLTs	0.000008	0.000004	0.0789	1 (1–1)

## Data Availability

Data can be obtained on reasonable request by contacting the corresponding author.

## Appendix A

**Table A1:** COVID-19 mortality in relation to demographics, comorbidities, disease severity, days in hospital and max FiO2 needs of the hospitalized patients.

Characteristics	Death (N = 74)	Survival (N = 432)	*p*-Value
Age			
Mean (SD)	84.9 (9.10)	64.6 (14.7)	<0.001 MW
Median [Min, Max]	88.0 [37.0, 96.0]	65.0 [22.0, 99.0]	
Gender			
Male	29 (39.2%)	233 (53.9%)	0.0264 x2
Female	45 (60.8%)	199 (46.1%)	
Disease Severity			
Critical	2 (2.7%)	13 (3.0%)	0.0327 fx
Severe	72 (97.3%)	383 (88.7%)	
Moderate	0 (0%)	34 (7.9%)	
Mild	0 (0%)	2 (0.5%)	
Days of hospitalization			
Mean (SD)	24.2 (12.7)	16.9 (7.98)	<0.001 MW
Median [Min, Max]	21.0 [6.00, 72.0]	15.0 [5.00, 59.0]	
FiO2			
Mean (SD)	76.3% (25.7%)	43.5% (18.6%)	<0.001 MW
Median [Min, Max]	90% [35%, 100%]	40% [21%, 100%]	
2-Level Disease Severity			
Severe/Critical	74 (100%)	396 (91.7%)	0.0197 x2
Mild/Moderate	0 (0%)	36 (8.3%)	
Comorbidities			
Cardiovascular disease (CVS)	41 (55.4%)	90 (20.8%)	<0.001 x2
Arterial Hypertension	33 (44.6%)	262 (60.6%)	0.0139 x2
Diabetes Mellitus	25 (33.8%)	71 (16.4%)	<0.001 x2
Malignancy	20 (27.0%)	20 (4.6%)	<0.001 x2
Immunosuppression	3 (4.1%)	4 (0.9%)	0.0679 fx
COPD	7 (9.5%)	31 (7.2%)	0.653 x2
Sleep Apnea	2 (2.7%)	4 (0.9%)	0.214 fx
Bronchial Asthma	1 (1.4%)	22 (5.1%)	0.227 fx
Chronic kidney Disease	4 (5.4%)	1 (0.2%)	0.00189 fx
Neurological Disease	29 (39.2%)	55 (12.7%)	<0.001 x2
Psychiatric Disease	8 (10.8%)	55 (12.7%)	0.786 x2
Morbid obesity	1 (1.4%)	11 (2.5%)	1 fx
Autoimmune Disease	1 (1.4%)	8 (1.9%)	1 fx
ECOG 4	10 (13.5%)	7 (1.6%)	<0.001 fx

DIC(ISTH) score ≥ 5: delineated to 22% of the deceased [65]. MW: Mann–Whitney U test, fx: Fisher exact test, x2: χ2 test for categorical variables, frequency of appearance and percentages% are registered.

**Table A2:** Cut-off values of lymphocytes, DD and CRP as for disease outcome, calculated from our study.

Mean absolute lymphocyte count	897/μL (AUC 0.808, sensitivity 0.743, specificity 0.733)
Nadir lymphocyte count	505/μL (AUC 0.838, sensitivity 0.757, specificity 0.756)
Percentage % of lymphocytes nadir	7.5% (AUC 0.823, sensitivity 78.6, specificity 73.1)
Mean DD	1.125 (mg/L) (AUC 0.828, sensitivity 73.6, specificity 72.7)
Zenith DD value	2.055 (mg/L) (AUC0.847, sensitivity 0.767, specificity 0.759)
Mean CRP value	45 (mg/L) (AUC 0.805, sensitivity 74, specificity 73.8)

**Table A3:** COVID-19 severity in relation to demographics, comorbidities, days in hospital, max needs of FiO2% and outcome.

Characteristics	Mild/Moderate (N = 36)	Severe/Critical (N = 475)	*p*-Value
Age	65.5 [37.0, 95.0]	68.0 [22.0, 99.0]	0.865 MW
Gender			
Male	9 (25.0%)	255 (53.7%)	0.00165 x2
Female	27 (75.0%)	220 (46.3%)	
FiO2			
Mean (SD)	22.3% (2.7%)	50.8% (22.8%)	<0.001 MW
Median [Min, Max]	21% [21%, 28%]	40% [21%, 100%]	
Comorbidities			
CVS disease	11 (30.6%)	121 (25.5%)	0.635 x2
Arterial hypertension	7 (19.4%)	208 (43.8%)	0.00742 x2
Diabetes Mellitus	4 (11.1%)	93 (19.6%)	0.304 x2
Malignancy	5 (13.9%)	37 (7.8%)	0.204 fx
Immunosuppression	0 (0%)	8 (1.7%)	1 fx
COPD	1 (2.8%)	38 (8.0%)	0.508 fx
Sleep Apnea	0 (0%)	6 (1.3%)	1 fx
Bronchial Asthma	0 (0%)	23 (4.8%)	0.395 fx
Chronic kidney Disease	0 (0%)	5 (1.1%)	1 fx
Neurological Disease	12 (33.3%)	74 (15.6%)	0.0119 x2
Psychiatric Disease	12 (33.3%)	51 (10.7%)	<0.001 fx
Morbid obesity	0 (0%)	12 (2.5%)	1 fx
Autoimmune Disease	2 (5.6%)	7 (1.5%)	0.127 fx
ECOG 4	0 (0%)	17 (3.6%)	0.623 fx
Days in hospital	14.5 [6.00, 48.0]	15.0 [5.00, 72.0]	0.646 MW
Outcome			
Improvement	36 (100%)	390 (82.1%)	0.0148 fx
Death	0 (0%)	74 (15.6%)	
Discharge with O_2_ support	0 (0%)	6 (1.3%)	
Death	0 (0%)	74 (15.6%)	0.0197 x2

MW: Mann–Whitney U test, fx: Fisher exact test, x2: χ2 test.
